# Collagen-derived dipeptide prolyl-hydroxyproline cooperates with Foxg1 to activate the PGC-1α promoter and induce brown adipocyte-like phenotype in rosiglitazone-treated C3H10T1/2 cells

**DOI:** 10.3389/fnut.2024.1375532

**Published:** 2024-05-15

**Authors:** Kaho Nomura, Yoshifumi Kimira, Ryosuke Kobayashi, Yuna Shiobara, Yoshihiro Osawa, Aya Kataoka-Matsushita, Jun Shimizu, Masahiro Wada, Hiroshi Mano

**Affiliations:** ^1^Department of Clinical Dietetics and Human Nutrition, Faculty of Pharmacy and Pharmaceutical Sciences, Josai University, Saitama, Japan; ^2^Department of Molecular Microbiology, Faculty of Life Science, Tokyo University of Agriculture, Tokyo, Japan; ^3^Nitta Gelatin Inc., Osaka, Japan

**Keywords:** obesity, collagen peptide, brown adipocyte differentiation, prolyl-hydroxyproline, PGC-1α, mitochondrial activity

## Abstract

**Background:**

The global obesity epidemic is a significant public health issue, often leading to metabolic disorders such as diabetes and cardiovascular diseases. Collagen peptides (CP) and their bioactive component, Prolyl-hydroxyproline (Pro-Hyp), have shown potential in reducing adipocyte size, with unclear mechanisms concerning brown adipocyte differentiation.

**Methods:**

We investigated the effects of Pro-Hyp on the differentiation of brown adipocytes in C3H10T1/2 mesenchymal stem cells, focusing on its impact on adipocyte size, gene expression related to brown fat function, and mitochondrial activity.

**Results:**

Pro-Hyp treatment decreased adipocyte size and upregulated brown fat-specific genes, including C/EBPα, PGC-1α, and UCP-1. Remarkably, it did not alter PPARγ expression. Pro-Hyp also elevated mitochondrial activity, suggesting enhanced brown adipocyte functionality. A Pro-Hyp responsive element was identified in the PGC-1α gene promoter, which facilitated the binding of the Foxg1 transcription factor, indicating a novel regulatory mechanism.

**Conclusion:**

Pro-Hyp promotes brown adipocyte differentiation, potentially offering a therapeutic strategy for obesity management. This study provides a molecular basis for the anti-obesity effects of CP, although further *in vivo* studies are needed to confirm these findings and to investigate the potential impact on beige adipocyte differentiation.

## 1 Introduction

Obesity is a serious global health threat due to its association with high risks of comorbidities such as type 2 diabetes mellitus and cardiovascular disease (1–3). Characterized by an abnormal increase in fat mass, this condition involves an increase in both the size (hypertrophy) and number (hyperplasia) of fat cells (4). The determinant of related complications is not the amount of fat itself but its localization and individual qualitative functional characteristics. Adipocytes can be classified into two types—white and brown—based on their origin, morphology, and function (5). When energy intake exceeds expenditure, the excess energy is stored in white adipocytes as lipids, particularly triglycerides (6). Brown adipocytes, containing numerous tiny lipid droplets and mitochondria rich in uncoupling protein 1 (UCP-1), can dissipate energy as heat in response to various stimuli such as cold exposure or pharmacological stimulations (7). Several key regulatory factors, including CCAAT/enhancer-binding protein alpha (C/EBPα), peroxisome proliferator-activated receptor gamma (PPARγ), and PPARγ coactivator-1 alpha (PGC-1α), are involved in the differentiation and activation of brown adipocytes (8, 9). Controlling the differentiation and activation of brown adipocytes and reducing lipids stored in white adipocytes is an attractive target for treating obesity and its associated metabolic abnormalities.

Collagen, an extracellular matrix protein, is characterized by at least one common Gly-X-Y repeating domain, with X and Y primarily being proline (Pro) and hydroxyproline (Hyp), respectively (10). Numerous bioactive peptides derived from collagen, containing Hyp in their sequences, are resistant to enzymatic degradation and are absorbed into the bloodstream as oligopeptides, exerting diverse physiological effects (11, 12). Continuous intake of collagen peptides (CP) has been reported to affect not only the morphology of fat cells but also serum lipid levels and the expression of genes related to lipid metabolism (13). Additionally, continuous administration of CP reduced testicular and visceral fat weight and decreased the size of fat cells in obese model rats (14). Prolyl-hydroxyproline (Pro-Hyp), particularly abundant in human blood following oral intake of CP, has been reported to reduce the size of cultured mature adipocytes (15). Recently, we reported that Pro-Hyp enhances the interaction between the Runx2 promoter and Foxg1, a forkhead box transcription factor, thereby promoting the transcription of Runx2 and stimulating osteoblast differentiation (16). Foxa3 and Foxo6, also forkhead box transcription factors, have been reported to regulate the transcription of PGC-1α by binding to the AT-rich motif in the PGC-1α promoter (17, 18). These results suggest that Pro-Hyp may regulate the differentiation of brown adipocytes by modulating the interaction between Foxg1 and the PGC-1α promoter. The adipocyte size reducing effect of Pro-Hyp could be due to the promotion of brown adipocyte differentiation. Therefore, CP and its active constituent, Pro-Hyp, may play a significant role in mitigating obesity. Nevertheless, the detailed mechanism by which Pro-Hyp controls the differentiation of brown adipocytes remains unknown. In this study, we investigated the effect of Pro-Hyp on the differentiation of brown adipocytes using C3H10T1/2 cells and explored its action mechanism.

## 2 Materials and methods

### 2.1 Cell culture and treatment

C3H10T1/2 cell line was obtained from the RIKEN Cell Bank (Tsukuba, Japan). C3H10T1/2 cells were plated in 6-well plate in Dulbecco’s Modified Eagle Medium (DMEM, Cat. #11885-084, Gibco, Thermo Fisher Scientific, MA, United States) containing 10% fetal bovine serum (FBS, Cat. #7524, Nichirei Biosciences, Tokyo, Japan) and 100 U/mL penicillin, and maintained in a humidified incubator at 37°C under a 5% CO_2_ atmosphere into a confluent. For the differentiation procedure, confluent C3H10T1/2 cells were induced to differentiate into adipocytes in a differentiation medium supplemented with 1 μM rosiglitazone (Cat. #R0106, Tokyo Chemical Industry, Tokyo, Japan) for 48 h. Rosiglitazone was dissolved in dimethyl sulfoxide (DMSO, Cat. #D8418, Sigma-Aldrich, St. Louis, MO, United States). 1 mM Pro-Hyp (Cat. #4001630, Bachem, Bubendorf, Switzerland) was diluted in the DMEM medium and co-treated with rosiglitazone for 48 h.

### 2.2 Oil Red O staining

C3H10T1/2 cells were fixed in 4% paraformaldehyde in phosphate-buffered saline (PBS, Cat. #D163-20145, Wako Pure Chemical Industries, Ltd., Osaka, Japan) for 20 min at room temperature. After fixation, the cells were washed twice with PBS and stained with Oil Red-O solution (Cat. #40491, Muto Pure Chemicals, Tokyo, Japan) for 20 min at room temperature. The cells were washed with running tap water to remove unbound stains. Then, it was visualized and photographed using a BZ-810 fluorescence microscope (Keyence, Osaka, Japan). At least 30 randomly selected images were collected for each treatment in each experiment. The cells stained with Oil Red O (i.e., adipocytes) were counted using BZ-H4M image analysis software (version 1.4.1.1, Keyence, Osaka, Japan).

### 2.3 RNA preparation and quantitative RT-PCR (qPCR)

The cells were cultured with or without Pro-Hyp and rosiglitazone for 4 days. Total RNA was extracted from the cells using an RNeasy Mini Kit (Cat. #74104, Qiagen, Hilden, Germany), and the first-strand cDNA was converted with a Prime Script Reagent Kit (Cat. #RR037A, Takara Bio Japan, Otsu, Japan). Then, a qPCR assay was performed with the Thermal Cycler Dice^®^ Real-Time System III (Takara Bio Japan, Otsu, Japan) using TB Green Fast qPCR Mix (Cat. #430STakara Bio Japan, Otsu, Japan). Glyceraldehyde 3-phosphate dehydrogenase (GAPDH) was used as the internal control for normalizing target gene expression, and their respective relative expression was analyzed by the 2 ΔΔCt method. The primers used are indicated in Table 1.

**Table 1:** Primer sequences for qPCR.

	Forward primer sequence	Reverse primer sequence
PGC-1α	ACA GCT TTC TGG GTG GAT T	TGA GGA CCG CTA GCA AGT TT
UCP-1	GGC ATT CAG AGG CAA ATC AGC T	CAA TGA ACA CTG CCA CAC CTC
PPARγ	ATC CAA GAC AAC CTG CTG CA	CGA TCT GCC TGA GGT CTG TC
C/EBPα	AGA ACA GCA ACG AGT ACC GG	TGG TCA ACT CCA GCA CCT TC
ACC1	GAT GGT TTG GCC TTT CAC AT	GAA GCC ACA GTG AAA TCT CG
FASN	GAG GAC ACT CAA GTG GCT GA	GTG AGG TTG CTG TCG TCT GT
GAPDH	ACT GAG CAA GAG AGG CCC TA	TGT GGG TGC AGC GAA CTT TA

### 2.4 Western blot assay

The cells were cultured with or without Pro-Hyp and rosiglitazone for 4 days. Cells were washed twice with ice-cold PBS and then lysed with RIPA Lysis Buffer (Cat. # 89900, Thermo Fisher Scientific, MA, United States) and containing a protease inhibitor cocktail (Cat. # 87786, Thermo Fisher Scientific, MA, United States). Cell lysates were centrifuged at 15,000 rpm for 30 min, and the supernatants were collected as protein samples. The protein concentration of each sample was measured with BCA Protein Assay Reagent (Cat. # 23227, Thermo Fisher Scientific, MA, United States). Proteins were separated by SDS-PAGE and transferred to PVDF membranes (Cat. #1704156, Bio-Rad, Hercules, CA, United States). After blocking with 5% skim milk in TBS-T consisting of 10 mM Tris-HCl (pH 7.4), 1.37 M NaCl, and 0.1% Tween 20 for 30 min at room temperature, the membranes were incubated with rabbit anti-UCP-1 (Cat. #14670, Cell Signaling Technology, Danvers, MA, United States), or rabbit anti-β-actin (Cat. #4970; Cell Signaling Technology, Danvers, MA, United States) 1/1000 diluted in blocking buffer for 1 h at room temperature. The membranes were washed with TBS-T and then incubated for 45 min at room temperature with an HRP-conjugated rabbit anti-mouse IgG (Cat. #7074; Cell Signaling Technology, Danvers, MA, United States) 1/1000 diluted in TBS-T. Labeled proteins were detected with EZ West Lumi plus (ATTO, Tokyo, Japan). Band intensities were determined using ImageJ software (National Institutes of Health, Bethesda, MD, United States).

### 2.5 Analysis of mitochondrial mass

Cells were incubated with MitoTracker red (Cat. #M7513, Thermo Fisher Scientific, Waltham, MA, United States; final concentration, 1 μM) for 1 h. Cells were washed with warmed PBS and fixed in 4% formaldehyde (Cat. #16120141, Wako Pure Chemical Industries, Ltd., Osaka, Japan). After stained with 4′,6-diamidino-2-phenylindole (DAPI, Cat. #S36964, Thermo Fisher Scientific, MA, United States), the cells were examined using a BZ-810 fluorescence microscope (Keyence, Osaka, Japan). Images were acquired using the same exposure time in each well. The experiment was performed in triplicate. Cell mitochondrial activity stained with MitoTracker Red was measured using BZ-H4M image analysis software (version 1.4.1.1, Keyence, Osaka, Japan).

### 2.6 Plasmid constructs

To clone DNA fragments from the −3,303 to −1 upstream region of the mouse PGC-1α gene into the luciferase reporter vectors, various lengths of DNA fragments were amplified by PCR using the genomic DNA extracted from the C3H10T1/2 cells as a template. The PCR products were cloned into the XhoI site of pGL3-control (Cat. #E1741, Promega, Madison, WI, United States) to generate the pGL3 PGC-1α promoter construct. The nucleotide sequences of the constructs were confirmed by DNA sequencing.

### 2.7 Luciferase reporter assay

The DNA transfections of 1 μg of each pGL3 PGC-1α promoter construct and 10 ng of pNL vector as an internal control vector (Promega, Madison, WI, United States) to the cells were performed by the Lipofectin method using Hily Max reagent (Dojindo, Kumamoto, Japan). The cells were incubated for 48 h before the transfection reagents were removed, and Rosiglitazone and Pro-Hyp were then added and cultured for 48 h. Luciferase assays were performed using the Dual-Glo Luciferase Assay System (Promega, Madison, WI, United States). After the Pro-Hyp treatments were completed, 80 μL of Dual-Glo reagent was added to each well, and then samples were incubated at room temperature for 10 min. The firefly luciferase luminescence was recorded using a Glo-Max Discover Microplate Reader with an integration time of 1 s. Eighty microliters of Dual-Glo “Stop & Glo” reagent was then added to each well and incubated at room temperature for 5 min before recording the NanoLuc luminescence. The firefly: NanoLuc luminescence ratio was calculated to determine the activity of the PGC-1α promoter.

### 2.8 Chromatin immunoprecipitation

We performed chromatin immunoprecipitation/quantitative polymerase chain reaction (ChIP/qPCR) analysis on C3H10T1/2 cells treated with and without 1 mM Pro-Hyp for 24 h to determine whether adipocyte differentiation transcription factors associated with the endogenous PGC-1α promoter *in vivo*. The ChIP experiment was performed to detect the effects of Pro-Hyp on the promoter activity of PGC-1α in the adipocytes. ChIP experiments were performed on C3H10T1/2 cells using the ChIP Assay Kit (Cat. #17-295, Millipore, Burlington, MA, United States), an anti-Foxo1 (Cat. #2880, Cell Signaling Technology, Danvers, MA, United States), antibody, an anti-Foxg1 antibody (Cat. # ab18259, Abcam, Cambridge, United Kingdom) and normal rabbit IgG (Cat. #2729, Cell Signaling Technology, Danvers, MA, United States), in accordance with the manufacturer’s instructions. The cells were cross-linked with 1% formaldehyde for 15 min at 37°C, washed, collected in cold PBS, lysed in SDS lysis buffer containing protease inhibitors, and sonicated to shear DNA. The sonicated samples were incubated with 2 μg of rabbit anti-Foxo1 antibody, rabbit anti-Foxg1 antibody, or normal rabbit IgG (Cat. #2729, Cell Signaling Technology, Danvers, MA, United States) overnight at 4°C. The antibody-protein-DNA complex was enriched by mixing with ChIP-grade protein A/G-agarose beads (Cat. #26161, Thermo Fisher Scientific, Waltham, MA, United States) for 1 h at 4°C. The bound DNA was purified following the instructions provided with the ChIP Assay Kit, amplified, and quantified using qPCR with specific primers (Table 2). Relative enrichment was calculated as the amount of amplified DNA relative to values obtained from Input.

**Table 2:** Primer sequences for ChIP.

	Primer sequence
P1	CAGTGTGAGCAAGTTAAGAT
P2	AGAGTCGATGTTTATTCATC
P3	TCATTATCAAGCAATCCTGG
P4	TAATGCAGAGTTATCTAGTG

### 2.9 Statistical analysis

The results are presented as the mean ± standard deviation (SD). The statistical significance of differences was analyzed using student’s *t*-test. A *p*-value of <0.05 was considered statistically significant.

## 3 Results

### 3.1 Pro-Hyp reduces the size of adipocytes

**Figure 1:**
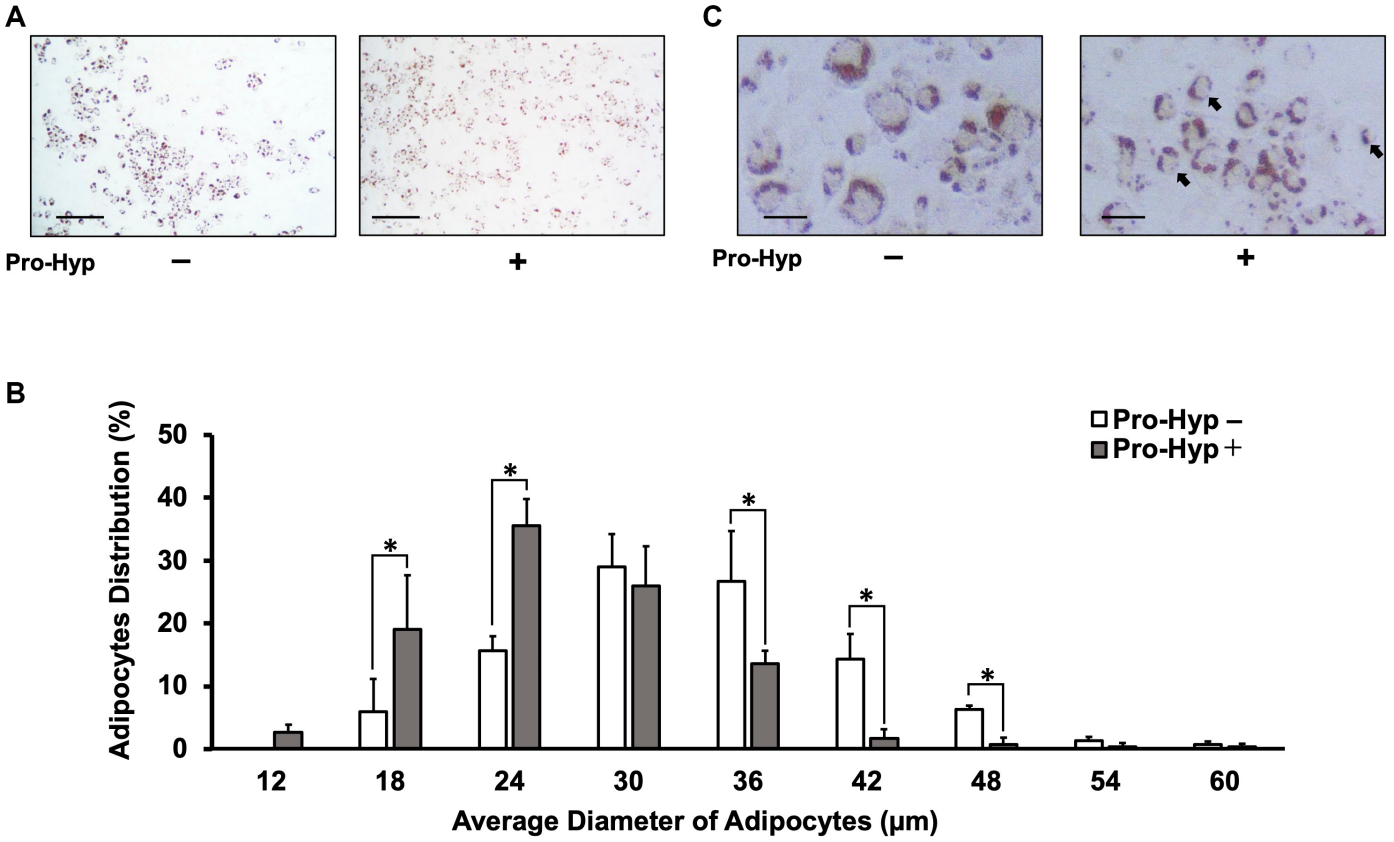
Pro-Hyp miniaturizes C3H10T1/2 adipocytes. C3H10T1/2 cells treated with 0.1 mM Pro-Hyp. Representative Oil Red O staining images of adipocytes. Scale bar, 250 μm **(A)**, 50 μm **(C)**. After 4 days of adipogenic differentiation, the cells were stained with Oil Red O. **(A,C)** Representative images of Oil Red O-stained adipocytes. C3H10T1/2 cells were treated with 0.1 mM Pro-Hyp. **(B)** The distribution of the average diameter of adipocytes. The diameter of adipocytes was evaluated using KEYENCE analysis software and ImageJ software. C3H10T1/2 was treated Pro-Hyp had a significantly miniaturized adipocyte diameter compared with untreated Pro-Hyp. Data are presented as the means ± standard deviation (*n* = 3), * vs. non-treated controls, *p* < 0.05.

The C3H10T1/2 cell line, a mesenchymal stem cell line, was used to study the effect of Pro-Hyp on adipocyte size. The differentiation of C3H10T1/2 preadipocytes was induced using the thiazolidinedione PPARγ agonist, rosiglitazone. To determine the impact of Pro-Hyp, cells were cultured for 5 days in the presence or absence of 0.1 mM Pro-Hyp and then stained with Oil Red O (Figures 1A,C). The results showed that adipocytes in the Pro-Hyp group had a smaller average size (24 μm) than the non-Pro-Hyp group. Additionally, a higher proportion of smaller adipocytes (12–24 μm) and a lower proportion of larger adipocytes (36–48 μm) were observed in the Pro-Hyp group (Figure 1B). These adipocytes also had smaller lipid droplets, characteristic of brown adipocytes (Figure 1C).

### 3.2 Pro-Hyp significantly increases mRNA expression of C/EBP, PGC-1α, and UCP-1 in C3H10T1/2 precursor adipocytes

**Figure 2:**
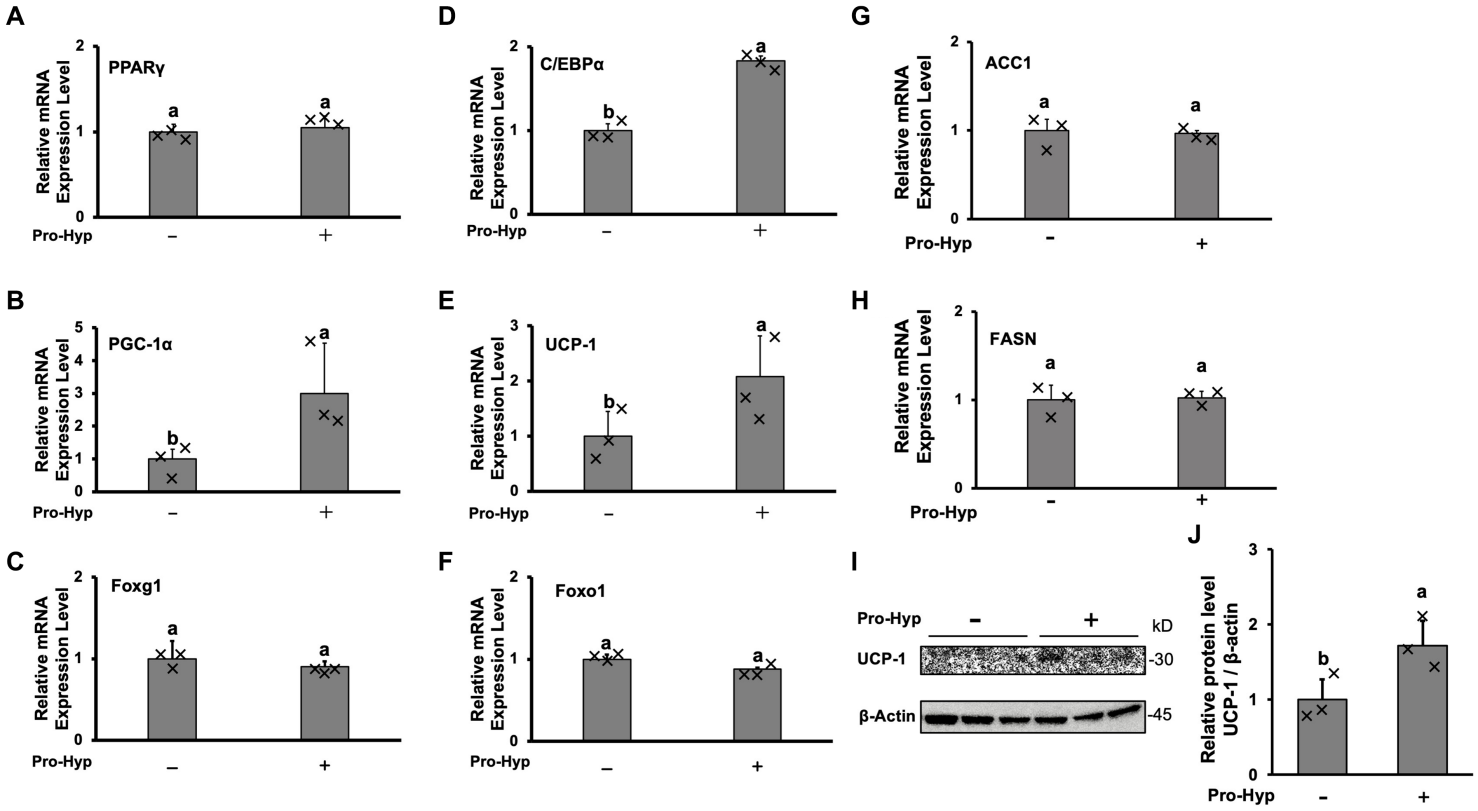
Pro-Hyp promotes brown adipocyte-linked gene expression. After culture for 4 days, the medium was replaced with an identical medium containing 0.1 mM Pro-Hyp. Cells were cultured with or without Pro-Hyp for 4 days. RT-PCR analysis of the mRNA expression levels of PPARγ **(A)**, PGC-1α **(B)**, Foxg1 **(C)**, C/EBP **(D)**, UCP-1 **(E)**, Foxo1 **(F)**, ACC1 **(G)**, FASN **(H)**. Results are expressed as relative values to GAPDH. Western blotting analyses **(I)** and quantification **(J)** of the expression of UCP-1. β-actin was used as a loading control. Data are presented as the means ± standard deviation (*n* = 3), * vs. non-treated controls, *p* < 0.05.

To explore the effect of Pro-Hyp on adipocyte size reduction, qPCR was conducted to measure the mRNA levels of genes associated with brown adipocyte differentiation and activation, namely PPARγ, C/EBPα, PGC-1α, and UCP-1. The results showed that, while PPARγ mRNA levels remained unchanged, Pro-Hyp significantly increased the expression of C/EBPα, PGC-1α, and UCP-1 mRNA (Figures 2A,B,D,E). The expression of transcription factors Foxo1 and Foxg1, crucial in gene regulation involving Pro-Hyp, remained unchanged with the addition of Pro-Hyp (Figures 2C,F). Pro-Hyp had no effect on the expression of ACC1 and FASN, which are involved in lipid synthesis or lysis (Figures 2G,H). Furthermore, Pro-Hyp treatment group also significantly increased UCP-1 protein expression (Figures 2I,J). These findings on adipocyte size reduction by Pro-Hyp suggest that Pro-Hyp is associated with up-regulation of genes related to brown adipocyte differentiation.

### 3.3 Pro-Hyp increases mitochondrial MitoTracker intensity in C3H10T1/2 precursor adipocytes

**Figure 3:**
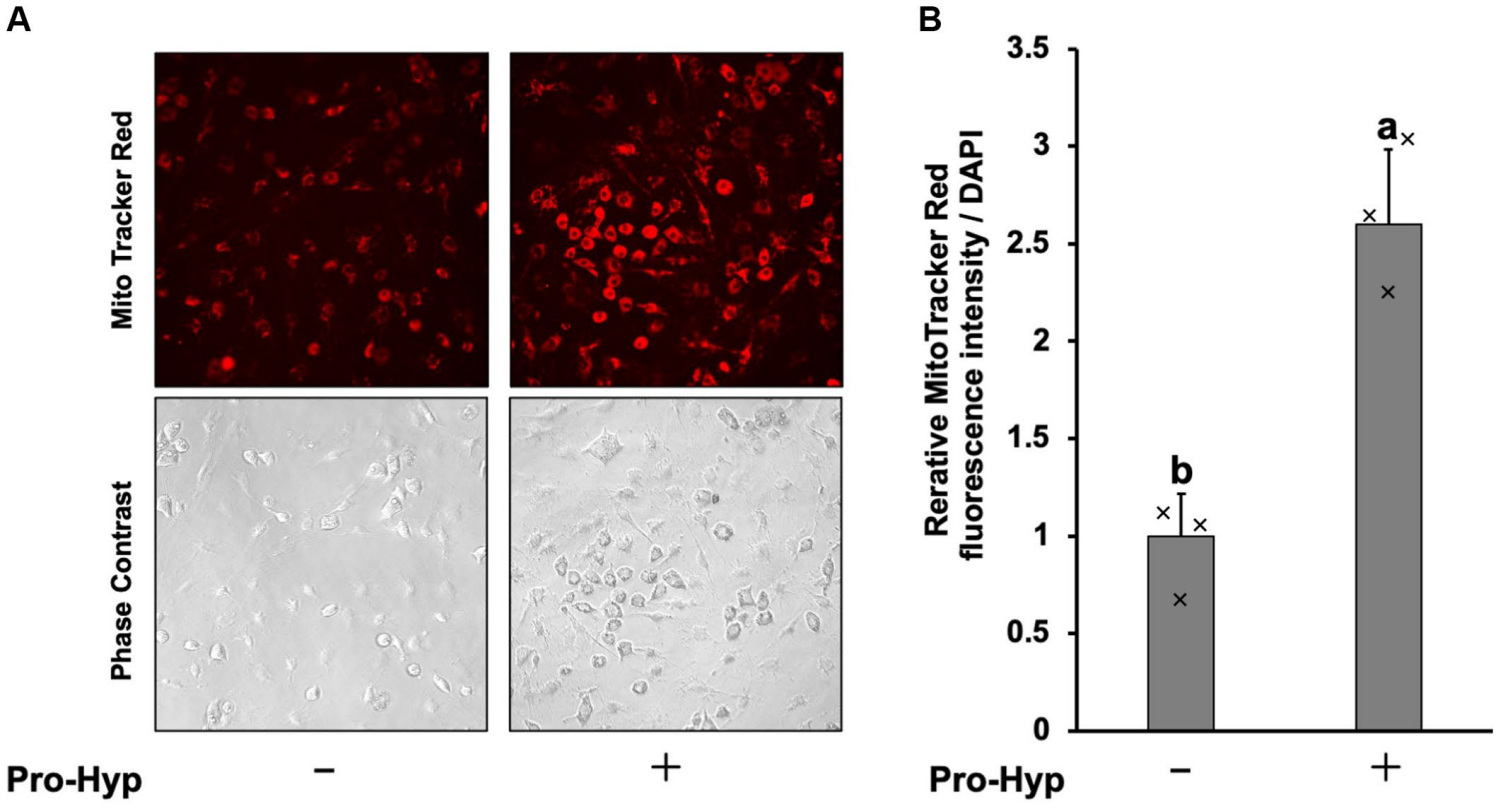
Pro-Hyp increased mitochondrial staining in C3H10T1/2 preadipocytes. **(A)** C3H10T1/2 cells were incubated. MitoTracker (final concentration 50 nM) was added to the culture medium without or without 0.1 mM Pro-Hyp, and fluorescence was observed with the same exposure time. Representative microscopy images are shown. **(B)** Quantification of images. Relative MitoTracker fluorescence intensity per DAPI was shown (*n* = 3). Data are presented as the means ± standard deviation (*n* = 3), * vs. non-treated controls, *p* < 0.05.

To assess whether Pro-Hyp stimulates mitochondrial mass, MitoTracker Red staining was used, showing an increase in mitochondrial fluorescence signal intensity (Figure 3A). Pro-Hyp treated C3H10T1/2 precursor adipocytes exhibited a significant increase in MitoTracker intensity compared to untreated cells (Figure 3B), indicating increased mitochondrial mass.

### 3.4 Pro-Hyp increases mitochondrial MitoTracker intensity in C3H10T1/2 precursor adipocytes

To assess whether Pro-Hyp stimulates mitochondrial activity, MitoTracker Red staining was used, showing an increase in mitochondrial fluorescence signal intensity (Figure 3A). Pro-Hyp treated C3H10T1/2 precursor adipocytes exhibited a significant increase in MitoTracker intensity compared to untreated cells (Figure 3B), indicating increased mitochondrial mass.

### 3.5 Identification of Pro-Hyp responsive elements in the upstream region of the PGC-1α gene

**Figure 4:**
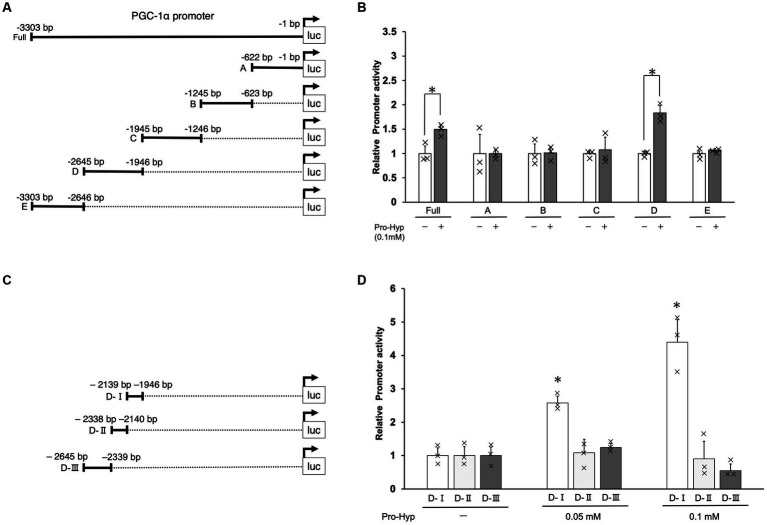
Identification of Pro-Hyp response element in the PGC-1α promoter. Schematic illustration of the luciferase expression vectors containing different regions of the PGC-1α promoter **(A,C)**. The C3H10T1/2 cells were transfected with 1 μg of pGL3-PGC-1α promoter DNA and 10 ng of pNL DNA. After 48 h, the cells were treated with Pro-Hyp for 48 h. The luciferase activity was measured and a Nano-Luc reporter vector, pNL was used as an internal control to normalize for transfection efficiency. Luciferase assay was used to identify the Pro-Hyp response element in the PGC-1α promoter **(B,D)**. Each data point is the mean ± S.D. of at least four independent assays. * vs. non-treated controls, *p* < 0.05.

To investigate whether Pro-Hyp induces differentiation of brown adipocyte-like cells by promoting the expression of PGC-1α, a key gene in this process, we conducted a series of experiments using the C3H10T1/2 cell line. Fragments of various lengths from the upstream region of the Pgc1α gene were cloned into luciferase reporter vectors to create constructs for luciferase reporter assays (Figures 4A,C). Specifically, the segments from −3,303 to −1 and the −2,645 to −1946 region (referred to as the D domain) upstream of the PGC-1α gene were tested. When cells transfected with these constructs were exposed to Pro-Hyp, they showed a significant increase in luciferase activity compared to when Pro-Hyp was absent, indicating a response to Pro-Hyp (Figure 4B). Further detailed examination using truncated mutants of the D-domain in the upstream of the PGC-1α gene revealed that the responsive region to Pro-Hyp is located between −2,139 and −1946 upstream of the mouse PGC-1α gene (Figure 4D). This finding is critical as it pinpoints the specific sequence within the upstream of the PGC-1α gene that responds to Pro-Hyp, suggesting a direct regulatory mechanism at the genetic level.

### 3.6 Pro-Hyp promotes Foxg1 binding to a Pro-Hyp responsive element upstream of the PGC-1α gene in adipocytes

**Figure 5:**
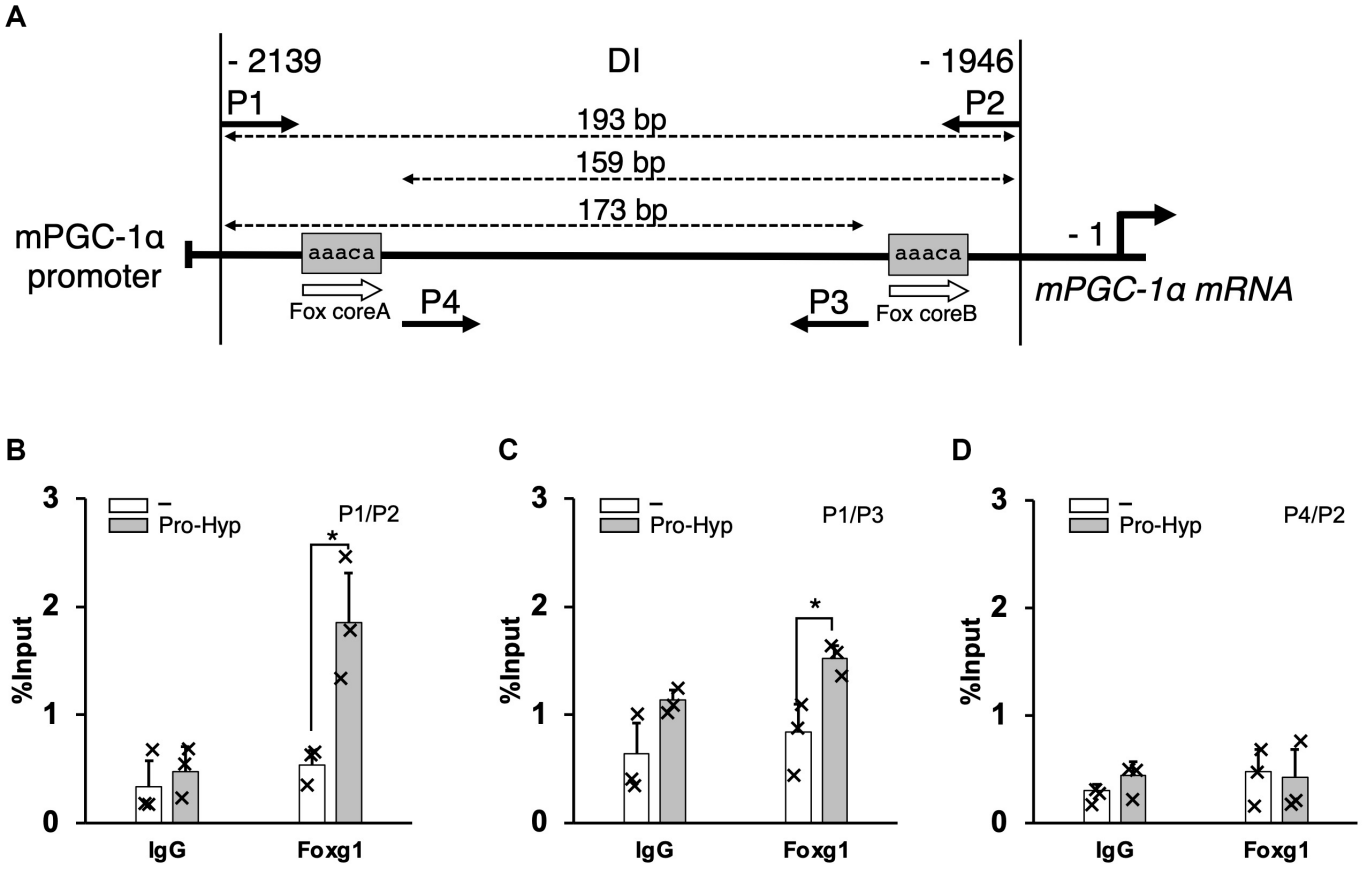
Pro-Hyp promotes the binding of Foxg1 to the PGC-1α promoter. Verification of the Pro-Hyp response element in the PGC-1α promoter, including the Fox-binding site. **(A)** Schematic representation of the relevant regions of the PGC promoter. P1–4 indicate the PCR primers used to perform ChIP/qPCR analysis. The positions of these primers and the size of the amplified fragments are shown at the top of the figure. **(B–D)** ChIP/qPCR analysis of the PGC-1α promoter in C3H10T1/2 cells. Cells were incubated with and without 1 mM Pro-Hyp for 48 h, followed by ChIP/qPCR analysis. Experiments were repeated three times with similar results. Data are presented as the mean + standard deviation (*n* = 3), *p < 0.05.

To further elucidate the mechanism by which Pro-Hyp influences brown adipocyte differentiation, we focused on the −2,139 to −1946 region upstream of the PGC-1α gene, identified as the DI region containing at least two the Fox core sequence. This specific region was studied to understand how the transcription factor Foxg1 and PGC-1α regulate Pro-Hyp-induced brown adipocyte differentiation. Chromatin immunoprecipitation (ChIP) followed by quantitative PCR (qPCR) was utilized to analyze the binding of Foxg1 to this region. The experiments were performed using primers specifically designed to target these sequences (as shown in Figure 5A and detailed in Table 2). In the presence of Pro-Hyp, Foxg1 was found to bind to the DI region upstream of the PGC-1α gene. This binding was notably absent in the absence of Pro-Hyp, underscoring the specificity of the interaction (Figure 5B). Particularly striking was the finding that, in the Pro-Hyp-treated samples, Foxg1 was significantly bound to the Fox core A sequence within the DI region (Figure 5C). In contrast, no significant binding to the Fox core B sequence was observed (Figure 5D). This highlights a targeted interaction between Foxg1 and a specific sequence within the DI region in response to Pro-Hyp.

Moreover, regardless of the presence of Pro-Hyp, no binding of Foxo1 to the DI region was detected, suggesting a unique role for Foxg1 in this regulatory process (data not shown). These results strongly suggest that Pro-Hyp facilitates the binding of Foxg1 to the specific Fox core A sequence upstream of the PGC-1α gene. This interaction appears to be a critical aspect of the regulatory mechanism by which Pro-Hyp influences gene expression related to the differentiation of brown adipocytes.

## 4 Discussion

Obesity is a common chronic disease resulting from an imbalance between energy intake and expenditure, leading to dysregulated lipogenesis. It poses a serious global health threat due to its association with many complications (19). Therapeutic strategies targeting the reduction of lipids stored in white adipocytes are effective. Still, approaches that enhance the activity of brown adipocytes, which can consume energy by producing heat, may offer promising new strategies for preventing and treating obesity. This is because the activity of these thermogenic adipocytes is reported to be inversely correlated with fat mass (20). Although drugs for obesity treatment are approved, strategies using natural compounds derived from food that do not cause side effects are still sought after. Recently, multiple reports have suggested the fat-reducing effects of collagen peptide intake (13, 14, 21). Pro-Hyp, an oligopeptide abundantly detected in human blood following collagen peptide intake, has been reported to reduce the size of fat cells (15). However, the detailed mechanism by which Pro-Hyp regulates brown adipocyte differentiation remains unknown. In this study, we investigated the effect of Pro-Hyp on the differentiation of brown adipocytes and its mechanism using C3H10T1/2 cells.

We treated C3H10T1/2 cells with the thiazolidinedione PPARγ agonist Rosiglitazone to induce adipocyte differentiation and examined the effects of Pro-Hyp on these adipocytes. Pro-Hyp was found to reduce the size of fat cells, suggesting its role in adipocyte miniaturization. Brown adipocytes, characterized by tiny multilocular lipid droplets and UCP-1 expression in the mitochondrial inner membrane, can dissipate energy as heat through uncoupling oxidative phosphorylation (22). Brown adipocyte differentiation is a complex process involving several factors, among which transcription factors like C/EBPα, PPARγ, and PGC-1α play crucial roles (8, 9). In our study, Pro-Hyp induced mRNA expression of C/EBPα but not PPARγ. Rosiglitazone, a PPARγ agonist, might have masked the effect of Pro-Hyp on PPARγ mRNA expression. Furthermore, Pro-Hyp induced mRNA expression of brown adipocyte-related genes PGC-1α and UCP-1 and increased mitochondrial mass. These findings are consistent with previous reports that Pro-Hyp increases mitochondrial area and UCP-1 in rat interstitial-vascular cells (15), suggesting that Pro-Hyp promotes brown adipocyte differentiation by controlling the gene expression of C/EBPα and PGC-1α.

PGC-1α, reported as a coactivator of PPAR-γ, regulates UCP-1 expression and thermogenesis in brown adipocytes. Given that Pro-Hyp is thought to promote UCP-1 expression by controlling PGC-1α expression, we conducted luciferase reporter assays using C3H10T1/2 cells to determine whether Pro-Hyp directly modulates mouse PGC-1α gene expression by regulating PGC-1α promoter activity. Pro-Hyp was found to promote transcription of PGC-1α. We also identified Pro-Hyp responsive elements upstream of nt −1946 and −2,139 in the PGC-1α gene.

Multiple reports have suggested that Pro-Hyp is involved in gene expression regulation in mesenchymal-derived cells (23–26). We previously reported that Pro-Hyp promotes Runx2 gene expression and osteoblast differentiation by facilitating the binding of Foxg1 to the Fox core sequence in the Runx2 promoter (16). It is conceivable that Pro-Hyp modulates the expression of target genes through Fox transcription factors. The Pro-Hyp responsive sequence we identified in the PGC-1α gene upstream of nt −1946 to −2,139 contains a Fox core sequence expected to bind forkhead box transcription factors, suggesting that Pro-Hyp could regulate PGC-1α gene expression through interactions with Foxg1 or Foxo1. Our study demonstrated using ChIP analysis that Pro-Hyp treatment facilitated the binding of Foxg1 to the PGC-1α gene upstream region containing the Fox core sequence between nt −1946 and −2,139. For Foxo1, no binding to the PGC-1α gene upstream region containing the Fox core sequence was observed. Binding to other members of the forkhead box protein family, FoxA3 and FoxO6, has not been confirmed. Foxg1 is a forkhead box protein known to be involved in neuronal differentiation and localizes to mitochondria (27). Overexpression of FOXG1 in nasopharyngeal carcinoma cells increased mitochondrial DNA copy number and ATP/ADP ratio, while knockdown had the opposite effect (28). These reports suggest that FOXG1 influences mitochondrial function. The increase in mitochondrial mass of C3H10T1/2 cells by Pro-Hyp in this study could be due to the upregulation of PGC-1α mediated by FOXG1.

In adipose tissue, fat cells are enveloped in a dense network of type I collagen. The expression of MMP-2 and MMP-9, which degrade extracellular matrix collagen, is thought to generate collagen metabolic products like Pro-Hyp (29). Fat cells differentiate and function in an environment rich in extracellular matrix (ECM) proteins (30). Our results may indicate that ECM collagen in adipose tissue is broken down and may promote beige adipogenesis. The reported fat-reducing effects of CP intake could be due to the action of CP containing Pro-Hyp and Pro-Hyp generated from collagen around adipose tissue, controlling adipocyte differentiation. Thus, Pro-Hyp could be an attractive target for obesity treatment.

## 5 Conclusion

While collagen peptides (CP) have been reported to exhibit anti-obesity effects, the mechanism by which CP induces brown adipocyte differentiation to exert these effects was previously unclear. Our study identified one of the molecular mechanism by which Pro-Hyp, a major collagen degradation product, promotes PGC-1α gene expression in brown adipocyte differentiation via Foxg1. However, further research is necessary to explore the potential impact of Pro-Hyp on beige adipocyte differentiation and its *in vivo* efficacy. Overall, our study reveals the potential importance of Pro-Hyp as an effective therapeutic target for obesity by promoting brown adipocyte differentiation.

## Data availability statement

The original contributions presented in the study are included in the article/supplementary material, further inquiries can be directed to the corresponding author.

## Ethics statement

Ethical approval was not required for the studies on animals in accordance with the local legislation and institutional requirements because only commercially available established cell lines were used.

## Author contributions

KN: Writing – review & editing, Writing – original draft, Investigation. YK: Writing – review & editing, Writing – original draft, Methodology. RK: Writing – review & editing, Investigation. YS: Writing – review & editing, Investigation. YO: Writing – review & editing, Investigation. AK-M: Writing – review & editing. JS: Writing – review & editing, Investigation. MW: Writing – review & editing. HM: Writing – review & editing, Visualization, Supervision, Conceptualization.
